# Integration of voltammetric analysis, protein electrophoresis and pH measurement for diagnosis of pleural effusions: a non-conventional diagnostic approach

**DOI:** 10.1038/s41598-020-71542-5

**Published:** 2020-09-16

**Authors:** Maria Elena Pipita, Marco Santonico, Giorgio Pennazza, Alessandro Zompanti, Sara Fazzina, Danilo Cavalieri, Francesca Bruno, Silvia Angeletti, Claudio Pedone, Raffaele Antonelli Incalzi

**Affiliations:** 1 Unit of Geriatrics, Campus Bio-Medico University of Rome, Via Alvaro del Portillo, 21, 00128 Rome, Italy; 2 Unit of Electronics For Sensor Systems, Department of Science and Technology for Humans and the Environment, Campus Bio-Medico University of Rome, Via Alvaro del Portillo, 21, 00128 Rome, Italy; 3 Unit of Electronics For Sensor Systems, Department of Engineering, Campus Bio-Medico University of Rome, Via Alvaro del Portillo, 21, 00128 Rome, Italy; 4 Pathology and Microbiology Laboratory, Campus Bio-Medico University of Rome, Via Alvaro del Portillo, 21, 00128 Rome, Italy

**Keywords:** Cancer screening, Cancer, Lung cancer, Non-small-cell lung cancer, Small-cell lung cancer

## Abstract

Pleural effusion is very common, but an etiologic diagnosis is often difficult. We used three unconventional diagnostic techniques (voltammetric analysis, protein electrophoresis and pH measurement) performed on pleural effusion to do a preliminary distinction between a neoplastic and a non-neoplastic origin. Pleural fluid samples were collected through thoracentesis, thoracoscopy, or post-surgery pleural drainage of 116 patients admitted to acute care wards. Samples were analyzed with the three unconventional techniques: voltammetric analysis using the BIONOTE system, capillary electrophoresis and pH measurement using a potentiometric method. The BIONOTE system is an innovative system that performs a cyclic voltammetric analysis of a biological liquid sample. The final output of the electrochemical analysis is an electrical pattern that represents a fingerprint of the analyzed sample and each sample has a different fingerprint. Data from the three unconventional diagnostic techniques were analyzed using partial least squares discriminant analysis to discriminate neoplastic from non-neoplastic effusions; we also evaluated sensitivity, specificity and percentage of correct classification. The mean age was 68 years (SD: 12); 78 (67.24%) participants were men. Results obtained from all the unconventional techniques employed showed that neoplastic and non-neoplastic pleural effusions were correctly classified in 80.2% of cases, with a sensitivity of 77% and specificity of 83%. The combined use of voltammetric analysis, protein electrophoresis and pH measurement of pleural fluid can easily and quickly distinguish a neoplastic from a non-neoplastic pleural effusion with reliable accuracy and represents an innovative diagnostic approach. In fact, this protocol can be executed in just few minutes directly in the patient's bed and it holds great promise to improve the prognosis and therapeutic chances.

## Introduction and background

Pleural effusion is very common and frequently requires a diagnostic thoracentesis^1^. In the diagnostic workup for pleural fluid, Light’s criteria represent the gold standard to distinguish a transudate from an exudate, but while their sensitivity is high, the specificity is quite low^2,3^. Currently, 15% of pleural effusions lack a definite diagnosis, despite invasive procedures like thoracoscopy and biopsy^2^. Thus, it would be highly desirable to test innovative diagnostic approaches.

In this paper, we explored three different approaches: a relatively new diagnostic technique (voltammetric analysis) and two established techniques (protein electrophoresis and pH measurement) that have rarely or never been investigated in pleural effusions.

Voltammetric analysis (VA) uses the multisensorial system BIONOTE^4^. This system has been used to measure different biological samples (urine, exudates collected from cutaneous ulcers) with promising results. VA can detect levels of uremic toxins^5^, urinary creatinine and the severity of renal insufficiency; it also allows to discriminate urine of healthy subjects from urine of patients with benign or malignant bladder^6^ and prostate tumors^7^. Furthermore, it has been confirmed that VA is a powerful technique to diagnose UTI^8^. Furthermore, BIONOTE analysis of the exudate on the ulcers of the lower limbs showed that lesions with different stages were associated with different voltammograms^9^.

Protein electrophoresis is commonly performed on blood, urine, and cerebrospinal fluid^10^. There are few data in medical literature about its usage on other biologic fluids, such as pleural effusions or ascites^11^. Light’s criteria evaluates proteins and lactic dehydrogenase (LDH) concentrations both in pleural fluid and in the blood to differentiate between an exudate and a transudate^11^, and it has been shown that adding protein electrophoresis to Light’s criteria could improve the diagnosis of a pleural effusion^12^. The authors argued that low weight molecules such as albumin and alpha1-antitrypsin can pass easily through the pleura in a transudation effusion; likewise, high weight molecules such as alpha-2 macroglobulin and some immunoglobulins are mostly found in exudative effusions due to an increase in capillary permeability^11,12^.

Finally, measurement of blood pH is really common to diagnose causes of pleural effusion but it has been demonstrated that investigation of pleural fluid pH holds evident theoretical premises. Indeed, normal pleural pH is 7.6, transudates’ pH is 7.4–7.55, whereas exudates’ pH is 7.3–7.45. A pH < 7.3 could be found in case of empyema, cancer, TBC or esophagus rupture^13^. Sometimes, pH > 7.6 is found in infections caused by Proteus species^14^. In case of neoplastic fluid, pH < 7.2 is a negative prognostic factor^15,16^.

Few available preliminary data suggest that each of these three diagnostic techniques could provide some information on the nature of pleural fluid. Thus, we performed a proof of concept study testing the ability of these approaches to achieve a preliminary distinction between the neoplastic and non-neoplastic origin of the pleural effusion and, thus, ease the following diagnostic pathway.

## Methods

### Study design

This was a 3-year observational prospective study carried out at “Campus Bio-Medico” Hospital in Rome (Italy). The data were collected from March 2016 to May 2019. The study protocol was approved by the Ethical Committee of Campus Bio-Medico of Rome University (protocol number: 31.16 TS ComEt CBM). Moreover, all methods were performed in accordance with the relevant guidelines and regulations in medical literature. All the participants were informed about all aspects of the trial and voluntarily confirmed their willingness to participate to the clinical trial for the advancement of medical research. A written informed consent was obtained from all participants and/or their legal guardians. Patients were recruited into the study from various departments of our hospital based on the following experienced clinical conditions: diagnostic or evacuative thoracentesis, diagnostic thoracoscopies, drainage positioning after lobectomies or cardiac surgery. There were no exclusion criteria.

In case of thoracentesis, samples were acquired and analyzed within 60 min; for thoracoscopy and post-surgical drainages, samples were collected within 24 h from drain placement and analyzed within 60 min. The time lag between samples collection and sample analysis is reported in Table 1.

**Table 1:** Time lag (hours) between sampling and sample analysis.

Time lag (hours)	Number of samples	Percentage (%)
0–1 h	67	57.76
1–6 h	28	24.14
> 6 h	21	18.10

The original protocol based on voltammetric analysis was lately integrated with electrophoresis and pH measurement. A total number of 196 patients had the voltammetric analysis performed but only 116 also had electrophoresis and pH measurement of pleural fluid performed.

The pleural fluid diagnostic works followed a standardized and validated procedure: the samples of pleural fluid underwent routine analyses (total cell count and differential formula, Gram staining and culture tests, Cytological analysis, Glucose concentration, Amylase dosage) and non-conventional analyses^17–20^. All the collected data have been registered in a Microsoft Excel database. The etiologic diagnosis of the pleural effusion was based on chemical–physical, microbiologic and cytologic examination^17–21^. Histological examination was performed just in case of pulmonary or pleural resection. Further diagnostic procedures were applied according to results obtained from routine clinical tests and individual clinical data.

Each sample was classified as neoplastic, infectious, inflammatory or cardiogenic. Having taken into account patient’s clinical history, we defined as inflammatory effusion the type of fluid that is not classified either as infectious neither as neoplastic nor as cardiogenic. Inflammatory effusions were mainly reactive following cardiac surgery.

Non-conventional analyses (BIONOTE, electrophoresis and pH measurement) were operated on pleural fluid before microbiological and cytological results available.

BIONOTE performs a cyclic-voltammetric analysis of the biological sample^4^ using commercial electrodes and a custom electronic interface^22^: it is based on an electrochemical cell with a three electrodes system (working electrode, reference electrode and counter electrode). A periodic voltage signal is applied between the reference and the working electrode: this potential interacts with the biological sample. Inside the sample, reactions will occur and specific currents will be generated according to the chemical composition of the sample. The currents flowing between the working electrode and the counter electrode will be registered and the final output of the electrochemical analysis will be an electrical pattern that represents a fingerprint of the analysed sample. Each sample has a different fingerprint.

The BIONOTE analysis was adapted to the measurement of the pleural fluid of patients for our study. 5 ml of pleural fluid were analysed at room temperature with BIONOTE without being pre-treated. The system applied a triangular voltage wave with a period of 100 s and a peak-to-peak voltage of 2 V to the sample^9^. The sensing system performed 5 cycles of voltammetry to obtain reproducible data; each cycle takes 100 s for a total of about 9 min. performing an average of all measuring cycles. 500 current values were acquired for each measurement: these values represented the fingerprint of the biological sample and had to be analysed using multivariate data analysis techniques.

The parameters for the acquisition, the number of samples and the sampling interval are controlled by a dedicated interface software.

**Figure 1:**
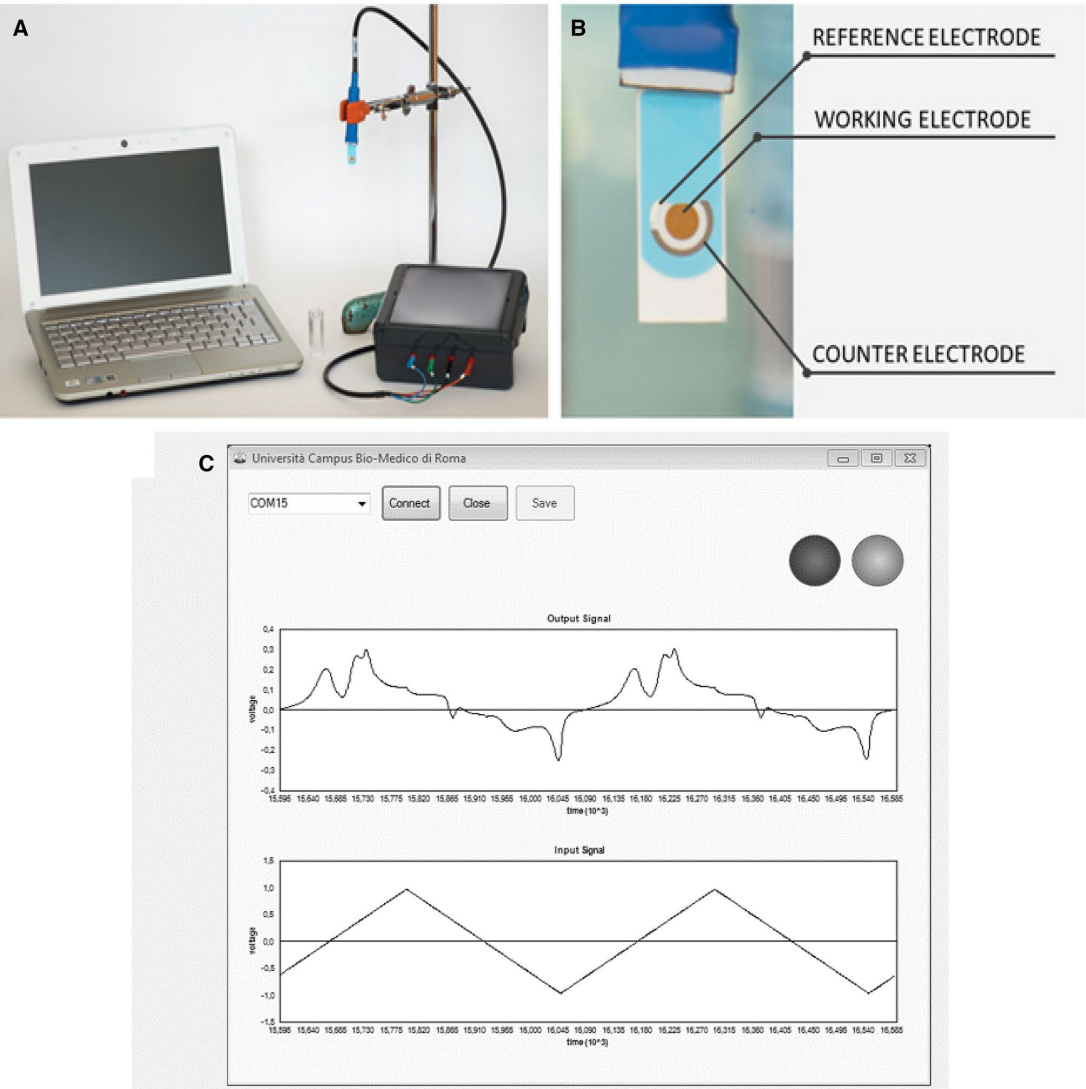
(**A**) The BIONOTE system consists of: an electronic interface connected to the personal computer with an USB cable; a power cable (12 V); a sensor cable; a disposable screen printed electrode; (**B**) The screen-printed electrode contains a reference electrode, a working electrode and a counter electrode; (**C**) Representation of data collected from the specific software as applied potentials and output signal voltages can be plotted in real time by a GUI with a specific software. This software was developed, using Visual Studio, by the Unit of Electronics for Sensor Systems, Campus Bio-medico University of Rome.

The BIONOTE System is shown in Fig. 1.

2 ml of the pleural fluid were used for pH measurement using a potentiometric method (GEM 4000 Premier Plus, Instrumentation Laboratory, IL, Werfen company Hartwell Road. Bedford, MA 01730 USA). Other 4 ml of biologic fluid were centrifuged at 3500 rpm for 5 min in order to perform capillary electrophoresis following the manufacturer’s instruction on Sebia Capillarys Protein E6 (Sebia, Lisses, Île-de-France).

### Statistical analysis

Data were analyzed through multivariate analysis techniques to assess whether non-conventional approaches could contribute to improve the diagnosis of neoplastic effusion in terms of diagnostic accuracy.

Partial Least Square Discriminant Analysis (PLS-DA) was applied on data obtained from voltammetry, on data obtained from the general characteristics of the population considered and on the clinical data of each patients (listed in the previous sub-section). PLS-DA is a learning machine algorithm often used in breath printing^23^: it is based on a linear analytical technique able to rotate the axes calculated in a direction where the least square of the classification error is minimal. PLS-DA provides a predictive model of two classes: neoplastic or non-neoplastic effusion. By the confusion matrix of the model it is possible to calculate the sensitivity and the specificity. CI (confidential intervals) considered were 99% for sensitivity and specificity.

## Results

### Characteristics of the analysed population

Clinical patients characteristics are reported in Table 2.

**Table 2:** Characteristics of the analysed population (SD: Standard deviation).

	Total (N = 116)	Neoplastic (N = 44)	Infectious (N = 10)	Inflammatory (N = 50)	Cardiogenic (N = 11)
**Etiology of pleural effusion**					
Sex (M) %	78	32 (41.0%)	5 (6.5%)	32 (41.0%)	9 (11.5%)
Sex (F) %	38	20 (52.6%)	4 (10.5%)	12 (31.6%)	2 (5.3%)
Total	116	52	9	44	11
Median age	68				
± SD	12				
**Pleural fluid extraction procedures**					
Drainage	12	4 (7.7%)	3 (33.3%)	4 (9.1%)	1 (9.1%)
Thoracoscopy	34	28 (53.8%)	2 (22.2%)	2 (4.5%)	2 (18.2%)
Thoracentesis	25	7 (13.5%)	3 (33.3%)	7 (15.9%)	8 (72.7%)
Post-surgical drainage	45	13 (25.0%)	1 (11.1%)	31 (70.5%)	0 (0.0%)

Mean age of the participants was 68 years (SD: 12); 78 (67.24%) were men. The etiologies of the pleural effusion were neoplastic (44.8%), infectious (7.8%), inflammatory (37.9%) and cardiogenic (9.5%). Pleural fluid sampling procedures tested were post-surgical drainage (38.8%), thoracoscopy (29.3%), thoracentesis (21.6%) and simple drainage (10.3%), positioned at the patient's bed without surgery.

Neoplastic effusions (N = 52) were associated with the following pathologies: pulmonary adenocarcinoma (which includes the histological diagnosis of poorly differentiated adenocarcinoma and metastatic pulmonary adenocarcinoma) (N = 25, 48.1%), pulmonary metastasis from other non-pulmonary malignancies (specifically carcinoma of the breast, colon and larynx) (N = 8, 15.4%), squamous cell carcinoma (N = 6, 11.5%), pleural mesothelioma (N = 2, 3.8%), other neoplasms (pulmonary neuroendocrine carcinoma and pulmonary epithelioid hemangioendothelioma) (N = 2, 3.8%) and detection of tumor cells on cytological examination but without specification of primary neoplasm (N = 9, 17.3%). On the other hand infectious effusions (N = 9), were mainly due to recent pneumonia in resolution (N = 5, 55.6%), active pneumonia (N = 3, 33.3%) or pleural empyema (N = 1, 11.1%).

Instead for inflammatory effusions (total N = 44), we noticed that most of the subjects (N = 33, 75%) relate to pleural effusions after cardiac surgery, while the extra 11 remained unexplained after naive drainage positioning (N = 3, 6.8%), thoracoscopy (N = 3, 6.8%) and thoracentesis (N = 5, 11.4%).

The post-cardiac surgery effusion were interpreted as reactive, except in case of microbiological isolation (in that case they were classified in the infectious group). Also, post-cardiac surgery effusions were classified as transudates when the drainage was needed for cardiac pleural effusion from heart failure after surgery. Finally, transudates (N = 11) largely derived from heart failure (N = 10, 90.9%), with only one case related to ascite decompensation.

### Results of voltammetric analysis on pleural fluid using BIONOTE, protein electrophoresis and pH measurement alone

In the subsample of 116 patients, we firstly evaluated the specific capacity of each individual techniques. The data collected from the 116 patients confirmed a good capacity for discrimination and a high sensitivity using BIONOTE alone, but a low sensitivity in discriminating between a neoplastic or non-neoplastic etiology. Analogously, both electrophoresis and pH measurement achieved satisfactory specificity, but a sensitivity of 55.8% and 48.1%, respectively (Table 3).

**Table 3:** Confusion matrix representing BIONOTE, electrophoresis and pH measurement on 116 patients.

	Neoplastic	Non-neoplastic
**BIONOTE analysis**		
Neoplastic	24	28
Non-neoplastic	7	57
**Protein electrophoresis analysis**		
Neoplastic	29	23
Non-neoplastic	18	46
**pH measurement**		
Neoplastic	25	27
Non-neoplastic	14	50

*BIONOTE* Percentage of correct classification: 69.8%. Sensitivity: 46.1%. Specificity: 89.1%.

*Protein Electrophoresis* Percentage of correct classification: 64.6%. Sensitivity: 55.8%. Specificity: 71.9%.

*pH measurement* Percentage of correct classification: 64%. Sensitivity: 48.1%. Specificity: 78.1%.

### Results of voltammetric analysis on pleural fluid using BIONOTE, protein electrophoresis and pH measurement in pairwise combination

We firstly combined two techniques (pH measurement and protein electrophoresis, BIONOTE and protein electrophoresis) and obtained a moderate improvement in the sensitivity.

Instead, the combination of BIONOTE and pH measurement, did not improve the diagnostic accuracy of individual components.

### Results of voltammetric analysis on pleural fluid using BIONOTE, protein electrophoresis and pH measurement in combination

**Figure 2:**
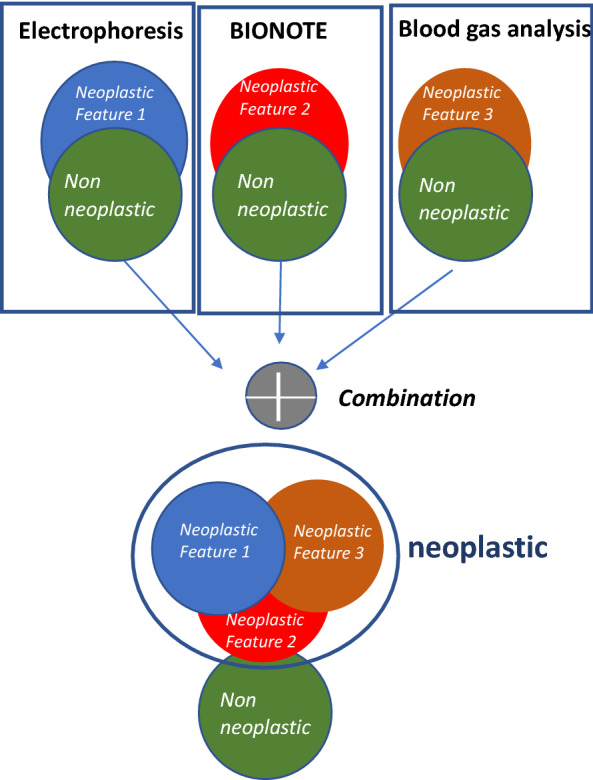
Rationale of the synergic model. Each of the three-measuring process shows a mild ability in the identification of neoplastic samples. Combination of all three techniques, i.e. exploring different features of the pleural fluid, improves the distinction between a neoplastic and non-neoplastic pleural effusion.

Finally, with the combination of all the three techniques (BIONOTE, pH measurement and protein electrophoresis) we significantly improved the classification of pleural effusion, achieving a percentage of 80.2% correct classifications, 80.2% sensitivity and 82.9% specificity. Figure 2 provides a conceptual graphic of how combining different low sensitivity approaches can result in a good sensitivity model (Table 4).

**Table 4:** Confusion matrices representing the combination of pH measurement and protein electrophoresis, the combination of BIONOTE and Protein electrophoresis and the combination of all three techniques.

	Neoplastic	Non-neoplastic
**Combination of pH measurement and protein electrophoresis**		
Neoplastic	35	17
Non-neoplastic	17	47
**Combination of Bionote and protein electrophoresis**		
Neoplastic	36	16
Non-neoplastic	10	54
**Combination of BIONOTE, protein electrophoresis and pH measurement**		
Neoplastic	40	12
Non-neoplastic	11	53

*pH measurement and protein electrophoresis* Percentage of correct classification: 70.6%. Sensitivity: 67.3%. Specificity: 73.4%.

*BIONOTE and Protein Electrophoresis* Percentage of correct classification: 77.6%. Sensitivity: 69.2%. Specificity: 84.4%.

*BIONOTE, pH measurement and Protein Electrophoresis* Percentage of correct classification: 80.2%. Sensitivity: 77%. Specificity: 82.9%.

## Discussion

The combination of BIONOTE, protein Electrophoresis and pH measurement of pleural fluid can easily and quickly distinguish a neoplastic from a non-neoplastic pleural effusion with an accuracy of 80%. Thus, this set of characteristic methods might improve the diagnostic pathway on pleural fluid. Interestingly, although individual techniques had poor sensitivity, their integration into a diagnostic set resulted in a satisfactory sensitivity. Indeed, comparably low levels of sensitivity might result from different combinations of true positives and false negatives and, thus, the integration of different low sensitivity techniques may yield a high sensitivity diagnostic set (Fig. 2).

In current medical practice, the diagnosis of a pleural effusion starts with the Light’s criteria, which allow to differentiate a pleural effusion in exudate or transudate. However, in case of exudates the Light’s criteria do not allow a definition of a neoplastic, inflammatory or infectious etiology. Therefore, clinicians must rely on a second level examination of pleural fluid for a definitive etiologic diagnosis: the cytological examination. Occasionally, lung biopsy and extra pulmonary diagnostic are needed to improve the diagnosis. However, these tests are time consuming and they carry a variable health risk and may be pricey. Thus, a fast-preliminary distinction of neoplastic from non-neoplastic effusions might optimize the diagnostic path, e g by sparing second level exams to patients if the initial etiology is consistent with non neoplastic. In high risk conditions, such as patients previously treated for cancer, the proposed method might achieve higher diagnostic accuracy and, thus, be qualified as first level exam of pleural effusions.

### Strengths and limitations of the study

Unfortunately, our study had some limitations. Firstly, 31 patients had reactive pleural effusions following cardiac surgery. While we planned this study to test our techniques in a very heterogeneous population, we later realized that it would have been more demonstrative to test the proposed set of data analysis in pleural effusions population. It is likely that retesting it in a pure and larger population of spontaneous effusions would disclose better clinimetric properties. Secondly, we performed the voltammetric analysis according to the methods previously developed for the diagnosis of skin and bladder infections^5–9^. However, this might not be the best option for identifying a neoplastic pleural effusion. Thus, efforts should be made to identify the best diagnostic procedure and, then, standardize it. Finally, in case of non-neoplastic pleural effusion, these three techniques were not able to predict whether the etiology is inflammatory, infectious or transudative.

On the other hand, our study did have some strengths. For instance, the innovative use of combining these three techniques together. Moreover, a primary distinction of the effusion etiology can be completed in a short time (about 30–40 min) without the need to carry out a blood test on the patient. In addition, a very small sample of pleural fluid (about 15–20 ml) is necessary to execute the analysis. These features are definitely advantageous allowing an initial classification of a patient with pleural effusion. Furthermore, our study was carried out using a heterogeneous population, which prevents the risk of overstating the diagnostic accuracy of the proposed method since it may occur in a convenient selected population. Finally, the BIONOTE can be easily transported and no specialized operator is needed to perform the analysis. Furthermore, the biologic sample should not be pre-treated and the model obtained could be stored in the memory of the device reinforcing the use of automatic classification for future clinical cases.

### Conclusions and future perspectives

The etiological diagnosis of a pleural effusion presently relies on conventional techniques which are time consuming and, do not consent to achieve a conclusive diagnosis in one out of 6 patients. Our pilot study has shown that an innovative approach which sees the integration of three low cost and fast diagnostic techniques allows to distinguish neoplastic from non-neoplastic pleural effusions with a very interesting accuracy. Present data refer to a pilot study and are biased by characteristics inherent to any pilot study as well as by the mix of medical and surgical effusions. Nevertheless, results seem robust enough to prompt confirmatory study, e g large scale and multicenter studies in order to verify how the observed results apply to the real life clinical practice. Furthermore, the tailoring of the voltammetric analysis to the diagnostic needs of the pleural effusion is expected to increase its diagnostic potential and, thus, of the whole set of methods. While traditional diagnostic methods will allow a compelling etiologic diagnosis, the proposed first level diagnostic approach might address selected diagnostic pathways based on the preliminary information on the nature of the pleural fluid.

## Abbreviations

VA: Voltammetric analysis
BIONOTE: BIOsensor-based multisensorial system for mimicking NOse, tongue and eyes
PLS-DA: Partial least square discriminant analysis
UTI: Urinary tract infections
CBM: Campus bio-medico
CI: Confidence intervals
GUI: Grafical user interface
